# An interactive Malaysian Childhood Healthy Lifestyle (i-MaCHeL) intervention programme to change weight-related behaviour in preschool child-parent dyads: Study protocol of a cluster randomised controlled trial

**DOI:** 10.1371/journal.pone.0276843

**Published:** 2022-10-31

**Authors:** Ahmad Faezi Rashid, Sharifah Wajihah Wafa, Ruzita Abd Talib, Nor Mazlina Abu Bakar

**Affiliations:** 1 School of Nutrition and Dietetics, Faculty of Health Sciences, Universiti Sultan Zainal Abidin, Kuala Nerus, Terengganu, Malaysia; 2 Faculty of Hospitality, Tourism, and Wellness, Universiti Malaysia Kelantan, Kota Bharu, Kelantan, Malaysia; 3 Faculty of Health Sciences, Universiti Kebangsaan Malaysia, Kuala Lumpur, Wilayah Persekutuan Kuala Lumpur, Malaysia; 4 Faculty of Business and Management, Universiti Sultan Zainal Abidin, Kuala Nerus, Terengganu, Malaysia; Wollo University, ETHIOPIA

## Abstract

**Introduction:**

Unhealthy weight, especially childhood obesity, is emerging as a growing epidemic and a challenge in developed and developing countries. Partnership with parents to promote healthy lifestyle changes may have a lifelong impact on weight-related outcomes in children. This study aims to determine the efficacy of an Interactive Malaysian Childhood Healthy Lifestyle (*i-MaCHeL*) intervention programme to change weight-related behaviour in preschool child-parent dyads.

**Materials and methods:**

The *i-MaCHeL* programme is a single-blind, theory-driven intervention, two-group cluster randomised controlled trial that evaluates the efficacy of a 3-month health promotion intervention in preschool child-parent dyads. In recognition of the value of multiple theoretical approaches, the strong theoretical basis consists of Social Cognitive Theory, Health Belief Model, and Trans-Theoretical Model principles underpinning the development of the intervention programme. In total, 460 child-parent dyads from 12 preschools in Terengganu, Malaysia, will be recruited. The children in the intervention group will expose to the *i-MaCHeL* classroom activities, while the parents will access the *i-MaCHeL* Web-based educational programme and numerous parent-child home-based online activities. The children in the control group will continue with any existing health-related activities, while the parents will receive the link to the general health newsletters. BMI z-score, dietary intake, physical activity, screen time duration, health-related quality of life, parental self-efficacy, parental role modelling, and parental policies will be assessed at baseline, 3 months’ post-baseline, and at 6 months’ follow-up (9 months’ post-baseline). General linear model repeated measure analysis will be used to determine differences between groups at the 3- and 9-month surveys with adjustment for potential covariates. Statistical analyses will follow intention-to-treat principles.

**Conclusion:**

We hypothesise that the combination of the classroom and interactive Web-based activities will have a strong potential to be effective strategies to sustain child-parent engagement and participation in the weight-related behaviour change programme.

**Clinical trial registration:**

**ClinicalTrials.gov Identifier:** NCT04711525.

## Introduction

In recent years, the prevalence of unhealthy weight in children has been alarming in many countries. Overall, in World Health Organization European Region, 28.7% of boys and 26.5% of girls (aged 6 to 9 years) were overweight (including obese), and 2.5% and 1.9%, respectively, were thin (COSI 2015–2017) [1]. In Malaysia, overweight and obesity coexist with thinness among children and adolescents. The National Health and Morbidity Surveys (NHMS) previously carried out in 2019 reported that 29.8% of children and adolescents aged 5 to 17 years were classified as either overweight or obese, and another 10% were classified as thin [2]. Furthermore, a growing trend toward the prevalence of childhood obesity has also been seen in Malaysia over the last few decades. The National Health and Morbidity Surveys (NHMS), previously carried out in 2011 and 2015, showed that the prevalence of obesity among children aged 5 to 9 years was increased from 6.7% in 2011 [3] to 14.8% in 2015 [4]. Thus, unhealthy nutritional status, especially childhood obesity, is considered one of the most significant public health issues in Malaysia.

Childhood obesity is a subject matter of priority worldwide. Childhood obesity results in physical and psychological comorbidities, which correlate with the chronicity of diseases that often persist into adulthood [5]. Early childhood-onset obesity is of particular concern because a study has reported that children who had a BMI between the 85th and 95th percentiles during their preschool years were more than four times as likely to become obese adults compared to their normal-weight peers [6]. Furthermore, another piece of evidence from a previous study has shown that overweight children have at least twice the risk of remaining overweight into their adult life compared to children in the healthy weight range [7]. Due to short-term and long-term impacts on health, including predisposition to diabetes and cardiovascular abnormalities, childhood obesity should be viewed as a serious concern for public health [8]. Moreover, the increase in the prevalence of childhood obesity and the tendency to become obese adults at a later age [9], highlighting this period is critical for preventive action [10]. Therefore, considering public health priority and currently available data on the nutritional status of preschool children, a comprehensive and effective weight-related behavioural intervention programme is urgently needed in Malaysia to reduce the risk of unhealthy weight, especially childhood obesity.

The preschool years are a vital time for establishing healthy lifestyle behaviours [11], and World Health Organization has highlighted it as a critical period for obesity prevention [12]. Systematic reviews of obesity prevention studies also suggested that preschool- and school-based interventions are useful in reducing the prevalence of childhood obesity [13, 14]. Preschool settings pose a realistic target for the health-related behaviour change intervention. The characteristics of the preschool, including policies and practices regarding dietary intake and physical activity, can significantly influence children’s healthy behaviour [15]. Moreover, preschools represent an ideal setting to address social inequalities because they provide access to the population and generally have the necessary facilities, curriculum, environment, and personnel to promote physical activity and healthy eating [16, 17].

Furthermore, preschool plays an essential role in facilitating or delivering health-related behaviour change intervention programmes. These include the organisational structure and the high level of trust that children and parents attach to preschool staff [18]. Also, preschool is a crucial stage for early education in Malaysian children under 18 years. The preschool years (aged 4 to 6 years) are the first stage of transition before starting primary school (aged 7 to 12 years), which is the compulsory requirement for education in Malaysia. In addition, declining physical activity, deterioration of dietary behaviours, and increasing recreational screen-time, increase during the early teenage years [17, 19]. Thus, the preschool represents an opportunity for delivering and exposing healthy lifestyle interventions before starting primary school. Besides that, the preschool setting is an arena where repeated exposure of education intervention can be implemented systematically and also can be a potential setting for influencing children’s lifestyles at an early age [20]. Therefore, weight-related behavioural interventions are particularly important in improving the physical activity and dietary behaviours of preschool children.

Moreover, children who participate in a lifestyle intervention at an earlier age (4 to 7 years) have greater weight loss over five years than those participating in the intervention at an older age (>11 years) [11]. Thus, weight-related behavioural interventions should start at an early age [9], and many studies have revealed greater success for programmes targeting the early-aged group [16]. During early childhood, lifestyle behaviours that promote obesity are just being learned, and it is easier to establish new behaviours than to change the existing ones [21]. Furthermore, health behaviours become more difficult to change with age and tend to track into adulthood but are quite malleable in the early years [22]. Once childhood obesity is established, it is difficult to reverse through interventions [13, 23]. Therefore, early childhood is an opportune time to intervene, and involving parents in interventions appears to be crucial [22].

The use of Information and Communication Technology (ICT) in adolescent and adult age groups has increased in recent years, especially in Malaysia. A report by the Department of Statistics Malaysia on ICT use and access by individuals and households survey has shown that the percentage of individuals in Malaysia aged 15 years and above that used the internet was 81.2% in 2018, which increased by 1.1% points as compared to 80.1% in 2017 [24]. In terms of ICT access by households, the percentage of households’ access to the internet increased by 1.3% points to 87.0% in 2018 as compared to 85.7% in 2017. The percentage of households’ access to mobile phones also increased by 0.1% points to 98.2% in 2018 as compared to 98.1% in 2017 [24]. The high ICT used and accessed by individuals and households in Malaysia highlights the importance of the web-based approach for effective home-based intervention programmes. In addition, it is well understood that online education provides many benefits for teaching and learning environments. Distributing information and materials online increases the availability of the intervention, making it easy for parents to find the information at their convenient time [20]. To our knowledge, there are limited intervention studies that integrate classroom activities and an interactive Web-based programme targeting preschool child-parent dyads, especially in Malaysia.

A finding from the recent systematic review revealed that most health-related interventions were concentrated in the primary and secondary school setting [25]. On the other hand, few intervention studies were conducted in the preschool or home settings. Thereby, the evidence for weight-related behavioural intervention primarily done in the preschool setting was relatively weak because of the few studies published [11, 14, 25]. Notably, most intervention studies to prevent childhood obesity in preschool settings were exclusively done in high-income developed countries [25]. Few were done in upper-middle-income developing countries. The systematic review study also suggested that the preschool-based randomised controlled trial combined diet and physical activity has been shown to have beneficial intervention effects [25]. However, all the preschool-based randomised controlled trials in the study were done in the United States of America (USA), which involved low-income or ethnic minority populations. Another systematic review study found that more than half of the health-related behaviour change intervention studies were conducted in the USA [26]. Most studies were done in developed countries, making it challenging to generalise the results globally. Considering the increasing trend of childhood obesity prevalence in developing countries, the lack of documented interventions is a serious limitation of the existing literature.

Interventions targeting multiple settings might hold the most promise in reducing the risk of unhealthy nutritional status, especially childhood obesity. The home environment is recognised as a strong influence on the health-related behaviours of children. Most school-based studies that included a home setting reported beneficial intervention effects [25]. However, evidence to support the effectiveness of the combination of preschool- and home-based intervention on weight-related behavioural intervention among children was limited [25]. Even though there are limitations to the available data, the importance of the home setting for weight-related behavioural intervention among children should not be discounted. Thus, based on current literature, more research with rigorous evaluation primarily done in preschool settings in combinations with home settings is greatly needed to support the development of comprehensive recommendations for weight-related behavioural intervention. Future research should expand on the existing evidence in preschool and home settings with specific attention to the implementation of studies in developing countries, especially in Malaysia, are warranted.

### Objectives

The general aim of this study is to evaluate the efficacy of the 3-month *i-MaCHeL* programme, a web-based, theory-driven intervention to change weight-related behaviour in preschool child-parent dyads. The primary objective is to compare the differences in BMI z-score between the intervention and control groups of preschool children at 3 months and 9 months’ post-baseline. The secondary objectives are: 1) to compare the difference in dietary intake, physical activity, screen time duration, and health-related quality of life between the intervention and control groups of preschool children at 3 months and 9 months’ post-baseline and; 2) to compare the difference in parental self-efficacy, parental role modelling, and parental policies between the intervention and control groups of parents at 3 months and 9 months’ post-baseline.

## Materials and methods

### Trial design

The Interactive Malaysian Childhood Healthy Lifestyle (*i-MaCHeL*) study is a single-blind, two-group cluster randomised controlled trial that evaluates the efficacy of a 3-month weight-related behaviours intervention in preschool child-parent dyads. The child-parent dyads will be randomised at the preschool level into either the intervention or control group at a 1:1 ratio. The present study protocol has been described according to the Standard Protocol Items: Recommendations for Interventional Trials (SPIRIT) 2013 statement [27, 28]. [Note: Completed SPIRIT checklist (S1 checklist) and TIDieR checklist (S2 checklist) were submitted along with the manuscript to the journal].

### Study setting

The present study will be conducted in the state of Terengganu, located on the East Coast of Peninsular Malaysia. There are eight districts of Terengganu, and this study will only include two districts: Kuala Terengganu and Kuala Nerus. These two districts are chosen based on demographic and logistic factors as they are within the study centre’s 35 miles (56 kilometres). Nevertheless, after careful consideration before selection, these two districts covered both urban and rural locations.

### Eligibility criteria

#### Eligibility (inclusion/exclusion criteria) of preschools

All preschools within Kuala Terengganu and Kuala Nerus districts will be eligible for inclusion. There are 71 preschools in the districts. Because of varying cluster size, the preschools with fewer than 26 children (minimum cluster size to be recruited) will be excluded from the study [10].

#### Eligibility (inclusion/exclusion criteria) of child-parent dyads

Within participating preschools, all children aged 5- and 6-year Malaysian citizens will be eligible for inclusion in the recruitment phase. The parent/guardian of the preschool children are eligible for the study if they could read and understand either English or Malay; are aged between 25 to 49 years; have regular internet access via a tablet device, mobile phone, or computer/laptop; have regular access to a phone with texting capability; have WhatsApp accounts or agree to create the accounts and; are comfortable to read/view materials on electronic devices. The age range of parents (with 5- or 6-year children) is between 25 to 49 years is applied in the present study to ensure that the parents are comfortable enroling in the fully online intervention.

Child-parent dyads will be excluded if the children are taking medications or have a medical condition with the potential to affect the weight or restrict age-appropriate play; the children have conditions that require the restriction of certain foods (e.g., celiac disease or food allergies) and; the parents suffer from a severe physical or psychological illness, making the study too demanding for the family.

### Sample size

The sample size is calculated for the primary outcome, BMI z-score. The sample size for individual randomisation of the present study is based on Formula 1 [29]. The values used in the sample size calculation are based on the BMI z-score data from Ahmad et al. (2018) [30] study, which is 1.95±0.45 and 2.09±0.35 for the treatment and control groups, respectively. Based on Formula 1, a calculated sample size required for each group arm is 131 child-parent dyads. It is calculated that, with at least 80% power to identify a small to medium effect size [31] at the 5% significance level, the present study needs a total of 262 child-parent dyads for the two-group arm.

$${}^{n}Individual\_Binary=\frac{{\left(z_{1-\frac{\alpha }{2}}+z_{1-\beta }\right)}^{2}[\sigma_{trt}^{2}+\frac{\sigma_{con}^{2}}{r}]}{\Delta^{2}}$$ (1)

**where**, $r=\frac{n_{con}}{\sigma_{con}},\Delta =\mu_{trt}-\mu_{con}$

*μ*_*trt*_ = Mean in treatment group = 1.95

*μ*_*con*_ = Mean in control group = 2.09

*σ*_*trt*_ = SD. in treatment group = 0.45

*σ*_*con*_ = SD. in control group = 0.35

*z* = Critical *z* value for a given Alpa (*α*) = 0.05 (two − sided) and Beta (*β*) = 0.2 with the value of $z_{1-\frac{\alpha }{2}}$ = 1.96 and *z*_1−*β*_ = 0.84

*r* = Ratio of the two sample sizes = 1/1

An adjustment using the design effect (DE) is made for the calculated sample size to allow for the design structure (due to the cluster sampling method), which results in larger sample sizes [32]. Taking into account unequal cluster size [10], assume the mean cluster size of 38 child-parent dyads will be recruited per preschool. Based on the Formula 2 [32, 33], given that the number of individuals per cluster (*n*) in the present study is 38 child-parent dyads and the Intra-cluster correlation coefficient (*ρ*) from the previous studies is 0.01 [18, 34], the calculated design effect of the present study is 1.37.


Designeffect(DE)=1+(n–1)ρ (2)

By considering the design effect is 1.37 and the sample size required for each group arm (^*n*^Individual_Binary) is 131 (see Formula 3), the total sample size of the individual randomisation is further increased from 262 to 360 child-parent dyads.


$$({}^{n}Individual\_Binary)\times Design\;effect\times 2$$ (3)

After estimating a loss to follow-up of 20% for this trial, the final sample size is increased to a total of 460 child-parent dyads (230 per group arm). Thus, based on the total number of participants in the two groups is 460 and the number of individuals per cluster is 38 child-parent dyads (see Formula 4), a calculated number of preschool clusters in the present study is 12 preschools. Therefore, 6 preschool clusters will be randomised to each experimental and control group.


$$n\_cluster=\frac{Total\;number\;of\;participants\;in\;the\;two\;groups}{number\;of\;individuals\;per\;cluster}$$ (4)

### Recruitment

#### Preschool recruitment

A list of the 71 preschools in Kuala Terengganu and Kuala Nerus will be obtained from the Terengganu State Education Department (the data is also available online at https://www.data.gov.my). After the screening for inclusion, 34 out of 71 preschools with less than 26 children will be excluded from the study. A list of 37 preschools will be eligible to participate in the present study. All the 37 eligible preschools will be assigned with a unique identification number and randomly ordered by an independent researcher using a random number generator software (Research Randomizer version 4.0) [35] to generate a list of random numbers. The eligible preschools will then be sequentially invited by email and phone call to achieve the target recruitment of 12 preschools. If the preschool decline to participate, the next preschool on the list will be approached. The first 12 preschools that agree to participate in the intervention programme will be enrolled in the study.

#### Child-parent dyads recruitment

**Fig 1:**
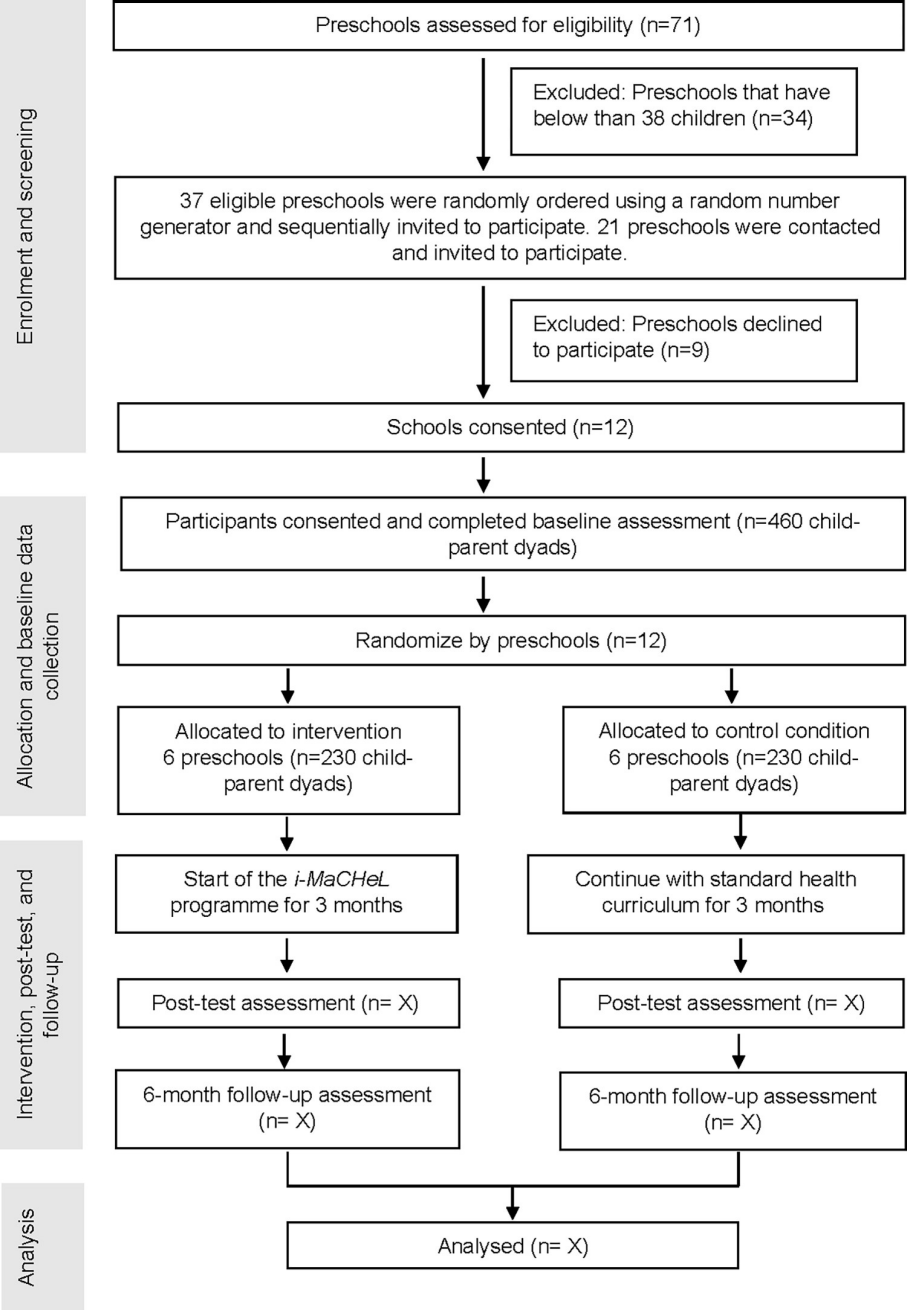
Flow chart of the *i-MaCHeL* programme trial design.

A list of children attending the included preschools will be obtained from the preschool teachers. All child-parent dyads in the preschools will be screened for eligibility criteria, and then consent to participate will be obtained from them. Teachers will distribute the information sheets and consent forms to preschool children to deliver to their parents. Information sheets and consent forms will have an explanation of the nature of the research, study components, and the requirements of the participants. Once consent forms are collected and eligible child-parent dyads are determined, the baseline data will be collected. After completing the baseline measurements, the child-parent dyads will finally be enrolled in the intervention programme. (see Fig 1 for study outline).

### Randomisation

The randomisation will be performed by an independent researcher who is blinded to the identity of the preschool, preschool personnel, or study participants [36]. The random assignment of the participating preschools (n = 12) to either the intervention or control group will be conducted using the block randomisation method. Using Research Randomizer (version 4.0) computer software [35], the 12 preschools will be divided into 6 blocks of 2 and then randomly assigned each preschool within a block to one of the two experimental conditions; condition 1 = control or condition 2 = intervention.

Randomisation will be performed at the level of preschools and not on the individual level to minimise contamination. The randomisation at the preschool level can minimise the possibility that the control group participants would influence the experimental group participants and vice-versa [37]. In addition, parents and children of the control group will not be informed about the existence of the intervention programme in other preschools. Since the recruitment of the participants occurs at the preschool level, it is a very low possibility that there will be spillover effects between the intervention and control groups. However, to avoid any possibility of “contamination” between the intervention group and the control group, participants will be asked not to share or discuss the intervention-based activities with others [38].

### Blinding

The single-blind RCT will be applied in the present study, in which the researcher but not the study participants know which intervention will be allocated at the preschool level. On the other hand, participants do not know to which group they will be assigned to improve external validity [39]. The single-blind method minimises bias and ensures that subjects’ knowledge of intervention assignments may not influence their response to the intervention programme [37].

### Intervention development

The information and contents (healthy eating, physical activity, and sedentary behaviour) of the *i-MaCHeL* programme are based on the current evidence-based national guidelines and recommendation for children: Malaysian Dietary Guidelines for Children and Adolescents 2013 [40] and Recommended Nutrient Intakes for Malaysia 2017 [41]. The contents also correspond to the information provided by the Nutrition Division of the Ministry of Health Malaysia (MOH) and the ten years long-term National Plan of Action for Nutrition of Malaysia III (NPANM III) 2016–2025 [42]. The intervention group is designed based on theory-based behaviour modification intervention. The intervention group will focus on the modification of TTM, SCT, and HBM constructs. The behaviour change strategies that will be employed in the intervention materials include: 1) SCT constructs which are role modelling, goal-setting, behavioural contracting and rewards, and self-efficacy; 2) HBM constructs which are perceived susceptibility, perceived severity, perceived benefits, and perceived barriers and; 3) TTM constructs which are decisional balance, and readiness to change [43–45].

In ensuring acceptability and responsiveness to the wants and needs of the participants, the content of the *i-MaCHeL* module and website has been extensively studied by involving a multidisciplinary panel of experts in paediatric obesity, nutrition, behavioural science, physical activity, multimedia, and preschool teachers. The multidisciplinary panel of experts has been consulted regularly throughout all phases of *i-MaCHeL* development to ensure that the most current knowledge and best practices are employed in the *i-MaCHeL* programme. The parents of preschool children are also involved in several steps of the *i-MaCHeL* module development. The most common questions, comments, feedback, and recommendations by the experts and parents are confirmed as the main topics to incorporate into the already planned intervention topics for the *i-MaCHeL* module. The planned intervention topics are changed in line with the data obtained.

The usability of the interactive website of the *i-MaCHeL* programme was pilot tested among a sample of parents having characteristics similar to the eligibility criteria of the present cluster RCT study but not in the RCT study. The study participants included 42 parents of preschool children. The parents were provided with online individualised feedback to allow parents to provide feedback, comments, and recommendation to improve the intervention process of the *i-MaCHeL* programme. The questionnaires to assess the usability of the *i-MaCHeL* website were adapted from the System Usability Scale (SUS) [46]. The SUS represents a simple ten-item instrument using a 5-point Likert scale. Descriptive analyses were used to assess the usability of the *i-MaCHeL* Website. The findings of the study showed that 83.3% of the Web users agreed that the programme website was not complicated, user-friendly, and not awkward to use. The programme website appeared satisfactory to users, especially related to the storyline, content, colour schemes, subtopic arrangement, and graphics.

### Intervention group description

The *i-MaCHeL* programme consists of the website component, interactive activities, teacher-led classroom activities, social media component, and theory-based behavioural change component. The parents in the intervention group will be conducted entirely online without face-to-face meetings. The parents will complete 13 modules over a 3 months’ period (one module per week). It takes approximately 60 minutes to complete each module. Below are detailed components of the *i-MaCHeL* intervention group.

#### Website component

To offer the best user experience, the *i-MaCHeL* website has been carefully designed to be interactive and user-friendly, in which the website is: 1) easily to navigate; 2) has clear, concise, and well-formatted content (utilise headings and subheadings to breakdown content, as well as short paragraphs and bulleted lists); 3) quick load-time; 4) responsive design and mobile-friendly (mobile-optimised website); 5) well organised information and; 6) good contrast colour scheme (proper contrast between the background of the website and content).

The website consists of infographics, reading materials, educational videos, and relevant pictures of a healthy lifestyle. The compilation of helpful guides and tips related to children’s healthy lifestyle, healthy recipes for kids, and a BMI calculator will also be provided on the website. In addition, the website also consists of interactive activities. Parents will be asked to provide regular input relevant to each module by sharing healthy meals/snacks pictures, active physical activities pictures, personal ideas/experiences relating to healthy lifestyle practices, and challenges to changing the unhealthy lifestyles of their children through padlet.com (Web 2.0 platform). The quiz and interactive games using Web 2.0 platforms (such as educaplay.com, quizizz.com, and jigsawplanet.com) will also be conducted online. Besides that, the website also consists of a module completion checklist. The checklist is an online review form that should be completed by parents upon the completion of every module. The checklist allows parents to determine if they have completed all the activities or assessments in the module. The parents will also be provided with online individualised feedback. The individualised feedback allows the parents to provide feedback, comments, and recommendations to improve the intervention process of the *i-MaCHeL* programme.

#### Classroom component (teacher-led classroom activities)

The teacher will deliver the classroom sessions in the preschool setting (with assistance and guidance from the researcher). Classroom activities focused on fun and active activities, exercises, and cooking demonstrations. The classroom activities will be conducted every two weeks in order not to overload the teachers. Each session takes approximately 60 minutes to complete. The preschool teachers will be encouraged to implement the activities during their teaching and learning sessions. The sessions at preschool will be conducted based on the date and time provided by the preschool teacher. Therefore, discussions with preschool teachers will be held in advance to ensure the delivery of the programme is aligned with the existing preschool teaching and learning process and the standard health education curriculum. The suitable time to conduct the programme will also be discussed with preschool teachers to ensure the programme can be implemented within the allocated time without interfering with the learning time of preschool children. The appropriate time based on the existing timetable, such as during outdoor activity class time (physical activity and free play), will be considered for the programme sessions.

#### Social media component (WhatsApp group)

The parents will be invited to join the official WhatsApp group of the intervention programme. On average, one message per day will be delivered in the WhatsApp group. The messages will include announcing the release of a new topic, a reminder to read the health information on the website, a reminder to participate in the online activities, and motivational text messages to promote programme involvement and participation.

Since the study is entirely conducted online among parents, there is limited opportunity to establish rapport with parents (compared to face-to-face or telephone-based interventions). Thus, the WhatsApp group is a convenient platform to facilitate communication and build rapport with and between parents. Parents will be informed that they can contact via text message (SMS/WhatsApp), email, or phone if they have questions or concerns at any time [22]. A WhatsApp group is accessible, where parents have the opportunity to communicate with other members and instructors. The WhatsApp group will be monitored daily to ensure the information discussed is consistent with evidence-based national guidelines.

#### Theory-based behavioural change component—self-monitoring

A Healthy Eating Stickers Book is developed to monitor and promote healthy food choices among preschool children. The sticker book is useful to help parents keep track of their child’s daily food intake for four weeks. Parents give a sticker to the child when the child consumes each food component in the sticker book (fruit, vegetable, fish, egg/chicken/meat, milk, and water). The Healthy Eating Stickers Book activities require active involvement from parents and children. The use of sticker books is cost-effective and has been used in successful interventions to change health behaviours within this age group [47].

Table 1 outlines the summary of the Interactive Malaysian Childhood Healthy Lifestyle (*i-MaCHeL*) programme components.

**Table 1:** Summary of the interactive Malaysian Childhood Healthy Lifestyle (*i-MaCHeL*) programme components.

Module theme	General objectives	Key components description	Targeted constructs	Activities
**Introduction**				
Module 1: Introduction to the *i-MaCHeL* Programme	to introduce the *i-MaCHeL* programme and set healthy lifestyle goals for children	General overview including objectives of the program, goal setting, contracting, readiness to change, information on WhatsApp group, and the weekly planner.	Readiness to change and Decisional balance (TTM); Goal setting, contracting, and rewards (SCT)	1. Parents share (online) barriers/challenges to changing the unhealthy lifestyles of their children. 2. Parents determine the level of readiness to change their children (online).
Module 2: Parents as Agents of Change	To educate parents to be role models for their children	Information on how parents can play a role in planning healthy eating and physical activity with their children.	Self-efficacy and role modelling (SCT); Action (TTM)	Parents share (online) a picture of a healthy lifestyle activity they do with their children.
Module 3: Healthy Weight	to explain the need of ensuring normal growth and weight for children’s health	Information on the importance of monitoring children’s growth and development; and how to determine children’s weight, height, and BMI status.	Perceived benefits and perceived barriers (HBM); Action (TTM)	1. Parents calculate their children’s BMI using an online BMI calculator. 2. Parents identify the BMI status using the BMI z-score chart.
**Physical activity components**				
Module 5: Active physical activity	to explain the need of keeping children to be physically active daily	Information on the importance of being physically active for children; and how to encourage children to stay active every day.	Perceived benefits and perceived barriers (HBM)	Parents ask children to complete an online quiz (memory game) to identify active physical activities.
Module 6: Sedentary Lifestyle	to explain the need of reducing sedentary activity for children	Information on the importance of limiting sedentary behaviour for children; adverse effects of using screen-based media devices for too long; and how to limit sedentary behaviour in children.	Perceived susceptibility and perceived severity (HBM)	Parents ask children to complete an online quiz (multiple choice answer) to identify sedentary behaviour.
**Healthy eating components**				
Module 4: My Healthy Plate	to explain the need of consuming food in a balanced, moderate, and varied for children	Information on how to choose healthy food based on the Malaysian Food Pyramid; to prepare meals based on the Malaysian Healthy Plate; and to determine the number of servings and portion sizes of food.	Perceived benefits and perceived barriers (HBM); Action (TTM)	Parents share (online) a picture of a healthy plate (Malaysian Healthy Plate) they served to children.
Module 7: Vegetables and Fruits	to explain the need of eating a variety of fruits and vegetables for children’s health	Information on the importance of eating vegetables and fruits every day; and how to provide/prepare a variety of vegetables and fruit every day for children.	Perceived benefits and perceived barriers (HBM); Self-monitoring (SCT)	1. Parents ask children to complete an online quiz (matching column game) to identify fruit and vegetables. 2. Parents monitor their child’s vegetables and fruit intake using sticker books.
Module 8: Whole Grains	to explain the need of eating whole grains for children’s health	Information on the importance of whole grain for health and disease prevention; and how to provide/prepare high-fiber wholegrain food for children.	Perceived benefits and perceived barriers (HBM); Action (TTM)	1. Parents share (online) pictures of healthy breakfast (whole grain-based food) they serve to children. 2. Parents ask children to complete an online quiz (multiple choice answer) to identify cereal-based foods.
Module 9: Food High in Protein	to explain the need of eating fish, meat, chicken, eggs, and legumes for children’s health	Information on the importance of protein-rich foods for children’s health; health risks of low intake of protein-rich foods; and how to provide/prepare healthy protein-rich food for children.	Perceived benefits and perceived barriers (HBM); Self-monitoring (SCT)	1. Parents ask children to complete an online quiz (memory game) to identify protein-based foods. 2. Parents monitor their child’s protein-rich foods intake using sticker books.
Module 10: Drinking Milk	to explain the need of consuming adequate milk and dairy products for children’s health	Information on the importance of milk and dairy products for children’s health; health risks of low intake of milk and milk-based products; and how to provide/prepare milk creatively for children.	Perceived benefits and perceived barriers (HBM); Self-monitoring (SCT)	1. Parents ask children to complete an online quiz (matching column game) to identify milk-based foods. 2. Parents monitor their child’s milk intake using sticker books.
Module 11: Fat, Do not Eat Too Much!	to explain the need of eating low-fat foods for children’s health	Information on the function of fat for body health; health risks of high intake of high-fat foods for children; and how to provide/ prepare low-fat foods for children.	Perceived susceptibility and perceived severity (HBM); Action (TTM)	1. Parents share (online) a picture of modified dishes to reduce the fat content in food. 2. Parents ask children to complete an online quiz (memory game) to identify foods high in fat.
Module 12: Beware Sugar and Salt in Food!	to explain the need of consuming foods low in sugar and salt for children’s health	Information on the health risks of high intake of food high in sugar and salt; how to provide/prepare low-sugar foods and beverages for children; and how to provide/ prepare less-salt foods for children.	Perceived susceptibility and perceived severity (HBM)	Parents ask children to complete an online quiz (multiple-choice answers) to identify foods high in sugar and salt.
Module 13: Refresh yourself with plain water	to explain the need of drinking plenty of plain water for children’s health	Information on the importance of drinking adequate plain water for health; health risks of water deficiency for children; and how to encourage the consumption of plain water for children.	Perceived benefits and perceived barriers (HBM); Self-monitoring (SCT)	1. Parents ask children to complete an online puzzle game. 2. Parents monitor their children’s water intake using sticker books.

Abbreviations: HBM: Health Belief Model; SCT: Social Cognitive Theory; TTM: Trans-Theoretical Model

### Control group description

The control group is designed based on the knowledge-based intervention (active control group). The control group programme contains general health information and is not tailored. The preschool children in the control group will not be exposed to the *i-MaCHeL* classroom activities, and their parents do not have access to the *i-MaCHeL* website materials. Thus, the children will continue with a standard health education curriculum only in the preschool setting, and their parents will receive general health newsletters via the WhatsApp group. The newsletters consist of general health information from the Ministry of Health Malaysia (MOH) official website. The website contains various knowledge-based topics related to nutrition, physical activity, and food safety. In ensuring that the intervention and control groups appear the same, the general health topics in the newsletters will have a look and feel similar to the *i-MaCHeL* group condition (nutrition and physical activity). The topic will also include other general health information relevant to the preschool life stage.

Generally, the parents in the control group will receive the same resources/information as in the intervention group; however, the information in the intervention group is designed to be more attractive and interactive in terms of audio-visual, text, and graphics. Besides, as opposed to theory-based behaviour modification, the control group programme will not focus on modifying TTM, SCT, and HBM constructs. In addition, the control group will not include interactive components (online activities and quizzes), goal setting, individualised feedback, and communication through the WhatsApp group, which have been designed to increase self-efficacy and improve the role modelling of the parents.

The parents in the control group will be invited to join the WhatsApp group. The Parents will receive messages through the WhatsApp group every week for three months. The messages are the announcement of the release of the newsletters, which direct them to various topics on the Ministry of Health Malaysia (MOH) official website. Unlike the WhatsApp group component in the intervention group, the establishment of the WhatsApp group in the control group is only to facilitate the delivery of information related to the programme. It is not intended for communication and building rapport with and between parents.

### Strategies to improve adherence to interventions

Parents will be provided with online completion forms that should be completed at the end of each module. Parents will note whether the sessions and activities in the module have been completed (yes/no). Besides that, the parents will be received weekly reminders and motivational text messages to promote programme involvement and adherence to the intervention programme. At the end of the programme, participants who complete all 13 modules will receive certificates. In addition, an end-of-module evaluation for each module will be conducted to obtain feedback, comments, and recommendation from parents to improve the module, website, and intervention process of the *i-MaCHeL* programme.

### Data collection

#### Outcomes

The primary outcome measure is the BMI z-score. The secondary outcomes measures are children’s dietary intake, physical activity, screen time duration, health-related quality of life, parental self-efficacy, parental role modelling, and parental policies. All the outcomes will be assessed at baseline, immediately following the intervention completion (3 months’ post-baseline), and at 6 months’ follow-up (9 months’ post-baseline) using anthropometric measurements (weight and height), three-day food record, and validated questionnaires. The participant’s timeline is presented in Table 2.

**Table 2:** Schedule of enrolment, interventions, and assessments in the *i-MaCHeL* programme. SPIRIT table as recommended by the 2013 SPIRIT Statement.

Activity & assessment	Study period								
	Enrolment	Allocation	Baseline	3-month intervention period			3-month post-baseline	6-month follow-up	Close-out
Timepoint	Oct 21—Mar 22	Apr 22	Month 0	Month 1	Month 2	Month 3	Month 3	Month 9	Jan 23
**Enrolment**									
Contact schools	x								
Eligibility screen	x								
Informed consent	x								
Allocation		x							
**Intervention & control**									
*i-MaCHeL* programme				x	x	x			
Control				x	x	x			
**Assessments**									
Sociodemographic			x						
BMI z-score			x				x	x	
Dietary intake			x				x	x	
Physical activity			x				x	x	
Screen time duration			x				x	x	
Health-related quality of life			x				x	x	
Parental self-efficacy			x				x	x	
Parental role modelling			x				x	x	
Parental policies			x				x	x	
Process evaluation							x		
**Statistical analysis**									x

### Sociodemographic and participant characteristics

Parents will be asked questions about their child’s participation. Parents will be asked to proxy-report general information about their family unit. Sociodemographic information of each child and his/her family will be collected by adapting questions in the general information section of the validated Preschool-age Physical Activity Questionnaire (Pre-PAQ) [48], which includes a child’s sex, ethnicity, date of birth, the number of children under 18 years old living in the household, the language spoke at home, household income, parents’ aged, education level, current working status, and current marital status. Household incomes will be categorised into 3 groups based on the income classification of Malaysia: Ringgit Malaysia (RM) 4,849 and less; RM 4,850 to RM 10,959 and; RM 10,960 and over [49]. The survey will be conducted at baseline only.

### Anthropometric measurements (BMI z-score)

Anthropometric measurements such as body weight and height will be assessed according to the standard procedures of the World Health Organization (1995) [50]. Each child will be asked to wear light clothing and no shoes during the measurements. Body weight and height will be measured to the nearest 0.1 kg and 0.1 cm, respectively [51], using an electronic SECA portable digital weighing scale (SECA 803, Hamburg, Germany) with an attached SECA stadiometer (SECA 217, Hamburg, Germany). All the measurements will be taken twice. However, if the height and weight measurements differ by more than 0.5 cm and 0.5 kg, respectively, a third measurement will be taken [22]. The measurements recorded for each participant are the mean values of the two closest measurements. Body Mass Index (BMI) will be calculated using the standard formula (kg/m^2^). BMI z-score curve based on the World Health Organization references will be used to classify weight status [52].

### Dietary intake assessment (three-day food record)

Dietary intake information of the children will be obtained from a three-day food record adapted from the Atlas of Food Exchanges & Portion Sizes [53] and the National Health and Morbidity Survey (2014) [4]. The three-day food record will be completed for three consecutive days (two weekdays and one weekend day) by their parents. The parents will be instructed to record all food and beverage items children consumed during the recording days, including the amount eaten (portion size), brand name, recipe, method of preparation, and place eaten.

From the values of the amount of food per day obtained from the three-day food record, the daily energy intakes will be determined using the Nutritionist Pro nutrient analysis software, version 5.2.0 (Axxya Systems; Nutritionist Pro, Stafford, TX, USA). The data from the food record will also be entered into the Malaysian Food Composition Database (MYFCD) website (http://myfcd.moh.gov.my/), which retrieves information from the national food composition database. The website is developed and continuously updated by the Nutrition Division, Ministry of Health Malaysia (MOH). The national food composition database contains standard food groups and dishes based on those available in the Malaysian population. The book Nutrient Composition of Malaysian Foods [54] and Atlas of Food Exchanges and Portion Sizes [53] will also be used to determine the nutrient composition. The nutrient information of food and formula products not available in the database will be sourced from websites, food companies, or the nutrient information panel on the product packaging. The serving size for each food group will be determined based on the recommended serving size in the Malaysian Dietary Guidelines for Children and Adolescents 2013 [40] and the Malaysian Dietary Guidelines 2020 [55].

#### Child physical activity and sedentary behaviours

Child physical activity duration (including light-, moderate-, and vigorous-intensity) and sedentary behaviour (screen time spent in front of a television, computer, tablet, or video game) will be assessed using the shorter version of the Preschool-age Physical Activity Questionnaire (Pre-PAQ), which is initially developed by Dwyer et al. (2011) [48]. The Pre-PAQ has acceptable validity and reliability in the preschool-aged children population. The Pre-PAQ is a parent-proxy report measure that provides a list of 24 different types of physical and sedentary activities [56]. It consists of questions about whether the children completed a specific type of physical activity. If so, the parents will provide information on how long this activity is completed across a weekday and weekends. The parents will be required to report on the time spent (in minutes) in the activities their child did "yesterday" and "last weekend". A 3-day mean for the activities will be calculated as an average of one weekday, Saturday, and Sunday [56]. The different kinds of physical activity are categorised into five levels: 1. Sedentary–no movement; 2. Sedentary play–limb or trunk moving; 3. Moving slowly; 4. Moving at a moderate pace and; 5. moving at a fast pace [48].

#### Health-related quality of life

The Health-Related Quality of Life (HRQOL) will be carried out using the validated Paediatric Quality of Life Inventory Version 4.0 (PedsQL) Generic Core Scales originally developed by Varni, Seid, and Kurtin (2001) [57]. The PedsQL 4.0 has acceptable validity and reliability in the preschool-aged children population. The PedsQL 4.0 is a parent-proxy report with 23 items to measure the two domains of HRQOL: physical (8 items) and psychosocial functions (15 items). The psychosocial functions include the emotional (5 items), social (5 items), and school functioning (5 items) functions of preschool children. A 5-point response scale is utilised (1 = never a problem; 5 = always a problem) [58].

#### Parental role modelling and parental policies

Home Environment Survey (HES) was developed and validated by Gattshall et al. (2008) [59] to assess parental role modelling and parental policies. The HES has acceptable validity and reliability among the parents of the preschool-aged children population. Parents will be asked about parental role modelling of the behaviours addressed in the intervention relating to their child’s nutrition and physical activity. The parental role modelling and parental policies of the Home Environment Survey (HES) consist of 18 parental role modelling questions (6 items of physical activity-related parental role modelling and 12 items of healthy eating-related parental role modelling) and 15 parental policies questions (5 items of physical activity-related parental policies and 10 items of healthy eating related parental policies). A 5-point Likert scale will be utilised for parental role modelling and parental policies items, with responses ranging from “never” (1) to “always” (5).

#### Parental self-efficacy

Parental Self-Efficacy for Healthy Dietary and Physical Activity Behaviours in Pre-schoolers Scale (PDAP) questionnaire was developed and validated by Bohman, Rasmussen, and Ghaderi (2016) [60] to assess parental self-efficacy relating to child’s healthy eating and physical activity behaviours. The PDAP questionnaire has acceptable validity and reliability among the parents of the preschool-aged children population [60]. An 11-point Likert scale will be used to record responses, ranging from 1 (not at all confident) to 11 (completely confident). Twelve of the 21 items from the questionnaire will be summed to yield separate scores for parental self-efficacy for promoting child physical activity. The other 9 items will be summed to yield scores for parental self-efficacy for promoting the child’s healthy eating. A higher score indicates a greater amount of parental self-efficacy [60].

All the previously validated questionnaires will be translated into Malay to suit the local setting and culturally adapted. Permission for translation and validation of all questionnaires will be obtained from the first original author. The questionnaires will be translated into Malay by two independent native Malaysian professional translators who are Malay speaking fluent in English. The translators carry out the necessary semantic and cultural-linguistic adjustments to obtain adequate correspondence in meaning [61]. The translations will be revised and reconciled to produce a forward-translated version of the questionnaires. Then, the Malay versions will be submitted to the three panels of experts to get the consensus that the Malay version demonstrates semantic and grammatical equivalence. The experts reviewed both versions, and after consensus, the instruments will be prepared in a single document. Each item of the questionnaires should be well understood and correctly interpreted by all parents. The adequacy of the format, presentation, clarity, and interpretation of each item and response option will be assessed. Verbal and written feedback on ease of completion, comprehension, and clarity of the Malay-translated questionnaires will be obtained from the panels. The researcher will revise and reconcile the translated questionnaires based on feedback and comments from the panels. A few modifications will be made to the wording accordingly [61].

The Malay version of the questionnaires will then be translated back into English by another two independent native Malaysian professional linguist experts fluent in English and Malay, blinded to the original version. Subsequently, the back translations will be compared to the original version of the questionnaires to ensure conceptual, semantic, and grammatical equivalence between the two versions. After final refinements are made, the questionnaires will be tested among parents of preschool children to determine the internal consistency reliability of the questionnaires. Cronbach’s alpha value of ≥ 0.70 is considered an acceptable value for the reliability of the questionnaires.

### Data management

The data generated in this study are quantitative data gathered through self-administered paper-based and web-based surveys, auto-generated website usage data, and anthropometric measures. In ensuring generated data are reliable, valid, and usable, the study used validated questionnaire items and standard procedures for the anthropometric data collection. Data collection, data cleaning, and statistical analyses will be performed by members of the research team. Study staff involved in data collection and management will be trained in implementing the procedures before and during data collection or management activities.

Once eligible child-parent dyads are determined, the children’s anthropometric data (BMI) and parent data will be collected within two weeks. Teachers will distribute the sealed envelopes to preschool children to deliver to their parents. The envelopes contain a set of three-day food record booklets and a child’s physical activity questionnaires. Parents will be asked to complete the questionnaires with their children throughout the study. After completing the surveys, the parents will return the completed food record booklet and questionnaires to preschools in sealed envelopes. The surveys will be kept by head teachers and subsequently collected by researchers. The researcher will check all completed forms for completeness and accuracy. When necessary, missing information will be requested and added during the parent’s visit to the preschool or by phone call. For the web-based surveys, the force step completion function will be used to prevent missing data, so informants can only continue with the questionnaire when all items are completed [62].

Data quality and maintenance of confidentiality will be regularly monitored by the supervisors. Outcomes of this data monitoring will be summarised in an overview and reported to all research personnel. During monthly study staff meetings, outcomes of this data monitoring will be summarised in an overview and reported to all research personnel [62]. Supervisors will regularly review data collection and management procedures at study staff meetings throughout the intervention period to ensure that they follow with fidelity and address any issues or barriers to implementation.

#### Data security and confidentiality

All data will be anonymised before entering data manually into an electronic data capture interface. All information will remain confidential and anonymised within the study team. Participant-specific data will be entered into the electronic data capture interface using only study IDs. Only the personal identifier of participants’ date of birth will be included in the database. In ensuring data quality and validity, the entered data will be double-checked by the person entering the data, and random checks will be performed regularly to ensure data validity. The statistical analyses will be conducted on anonymised data.

To anonymise the data, each participant will be assigned with a unique identification number (ID number), and no participant identifying information will be stored alongside data. The ID number with personal information will be safely stored separately from the trial data in order to secure confidentiality. Consent forms will be stored separately from participant data. The ID number will be used to administer and track all data collection, stored separately from data collection tools and locked in a secure room with limited access permissions. In ensuring the long-term preservation of the data generated in this study, the data files will be stored on secure password-protected university servers that are backed up daily, and access is restricted to researchers with passwords. Data generated via online survey software will be downloaded in spreadsheet format at least twice weekly. Data will be checked regularly to ensure the accuracy of data capture. All data will be uploaded to the password-protected university servers.

### Statistical methods

Differences in changes over time between the intervention and comparison groups will be assessed for each outcome. General linear model repeated measure analysis will be used to determine differences between groups over time (baseline, 3-month, and 9-month) with adjustment for potential covariates. Intention-to-treat (ITT) principles will be applied for parametric data. All participants will be analysed in both intervention and control groups regardless of whether they attended all data collection time points or completed the intervention [22]. The intention-to-treat principle is useful to preserve the sample size by including all randomised respondents regardless of noncompliance or withdrawal. The principle will be used to lower the probability of reduced statistical power due to the dropouts excluded from the final analysis.

Descriptive statistics (percentages or mean ± SD) will be calculated to describe participants’ sociodemographic characteristics and scores for each outcome measured by treatment and control groups. Repeated measures Analysis of Covariance (ANCOVA) will be performed to determine significant differences between groups at the 3- and 9-month surveys. The measures analyses will be controlled for covariate variables known to influence the outcome measures (child gender, parent education level, parent age, and parent income) [22]. The significance level will be set at ≤ 0.05. Analyses will be performed using Statistical Product and Service Solutions (SPSS; version 25.0; IBM, Armonk, NY, USA).

As randomisation will be at the school (cluster) level, the primary analysis will be adjusted for the clustering effect and the baseline value of the outcomes [63, 64]. Subgroup analysis will be performed to determine the direction of intervention effects by socioeconomic status, sex, and weight status. Where appropriate, multiple imputation methods will be used to account for missing data [18].

### Ethical considerations

Ethical approval was obtained from the Universiti Sultan Zainal Abidin Human Research Ethics Committee, Malaysia, on August 24, 2020, with reference No. UniSZA/UHREC/2020/184. The ethics committees approved the consent form, information letter, and study consent procedure. The study protocol was registered with ClinicalTrials.gov on January 15, 2021 (Identifier No: NCT04711525). Any amendments to the study protocol will be submitted for ethical approval prior to implementation. Written informed consent will be obtained from all parents/caregivers prior to the commencement of the study. Verbal assent will be sought from children prior to their enrolment in the study [47]. The participants will be informed and shall receive an explanation regarding the purpose of the study. The participants are given the right to refuse or not to participate in the study. They will then be given the consent form during the baseline visit. After signing the form, a copy of the signed consent form will be returned to the respective participants and kept for their records. No financial compensation will be provided for all participants.

Adverse events will be monitored, reported, and handled appropriately. Existing studies on the weight-related behavioural intervention of child-parent dyads provide no evidence of any associated risks, deterioration, or complications. Even though the possible adverse events directly related to the intervention are low, all adverse events reported spontaneously by the participant or observed by the researcher or mindfulness preschool teachers will be documented and reported to the relevant research review boards within 24 hours. Adverse events include emotional distress caused by a trial procedure (either the outcome assessments or the intervention delivery). Participants are entitled to withdraw from the study at any point and do so with no disadvantage. Participation in the study may be discontinued at all times with no obligation to provide a reason.

### Significance and outlook

Considering the severe consequences of paediatric obesity and the high likelihood of becoming obese in adulthood [65], there is a strong rationale for focusing, planning, and evaluating the weight-related behaviour intervention programme among preschool-aged children. The present study aims to assess the efficacy of the *i-MaCHeL* programme for preschool child-parent dyads. The *i-MaCHeL* is a theory-guided, 3-month programme developed to educate child-parent dyads in encouraging healthy lifestyle behaviour among preschool children. The *i-MaCHeL* programme will target multifactorial weight-related behaviour, which are diet, physical activity, and sedentary behaviour.

The National Plan of Action for Nutrition of Malaysia III (NPANM III) 2016–2025 is a ten years plan for addressing food and nutrition challenges in Malaysia [42]. The NPANM III has also focused on promoting healthy eating and active living for Malaysian communities. Various activities related to the school setting have been identified under the NPANM III to be implemented at the national level by the Ministry of Health Malaysia (MOH) and the Ministry of Education Malaysia (MOE) agencies. The activities such as reviewing curriculum specifically for healthy eating components, developing healthy eating educational tools, and involving parents in promoting healthy eating have been identified as effective strategies under the NPANM III. Therefore, the development of the *i-MaCHeL* programme is in line with the framework of NPANM III. *The i-MaCHeL* focuses on early childhood intervention and empowering the role of parents as gatekeepers of their child’s diet and physical activity. If found to be efficacious, the *i-MaCHeL* programme may have the potential to be a new programme for the Ministry of Education Malaysia (MOE) to assist preschool teachers in promoting a healthy lifestyle in early childhood education. Besides that, the *i-MaCHeL* programme may serve as a guideline in developing suitable recommendations and nutritional interventions for communities, which may help reduce the burden of the Ministry of Health Malaysia (MOH) to overcome the complications of obesity.

The *i-MaCHeL* programme is designed with an innovative approach to delivering a health-related behaviour change programme among child-parent dyads to prevent malnutrition risk among children. The *i-MaCHeL* programme encourages children to practice a healthy lifestyle and educates parents to shape home lifestyles for optimal child growth and development. The combinations of the two modes of delivery (classroom activities and the interactive Web-based programme) will have a strong potential to be effective strategies to sustain child-parent engagement and participation in the health-related behaviour change programme. The *i-MaCHeL* programme is unique, given its Web-based approach provides an opportunity for self-paced learning, in which parents can access the *i-MaCHeL* website anytime. Moreover, the present study will contribute to the literature on online interventions for childhood malnutrition prevention, where parents are the agent of change. The findings of this study will also enhance the understanding of how parents access, use, collaborate with others, and engage in a Web-based intervention. The essential components and features of the *i-MaCHeL* programme are believed to fill the current gaps and align with the current recommendations of the childhood malnutrition intervention studies, which may lead to a more substantial impact on reducing the double burden of malnutrition in children, especially in Malaysia.

### Dissemination plans

Communication and dissemination activities will be focused on the organisations engaged in guidelines on the management of nutrition and physical activity in children, such as the Child Nutrition Sector and Nutrition Department of the Ministry of Health Malaysia and the general public. The findings of our study will be published in appropriate peer-reviewed academic journals. The findings of the study will be presented at scientific conferences.

### Plans to give access to full protocol, participant level-data, and statistical code

There are currently no plans to allow public access to the full protocol, participant-level data set, or the statistical code. Nonetheless, this will be supported by the project management committee if the researchers want to access the data set (e.g., to perform a secondary analysis or meta-analysis).

## Supporting information

S1 Checklist SPIRIT checklist. (DOC)

S2 Checklist TIDieR checklist. (DOC)
